# Controlled-release urea enhances nitrogen fertilizer use efficiency by synchronizing soil nitrogen supply with nutrient uptake in Oilseed Rape

**DOI:** 10.3389/fpls.2026.1851107

**Published:** 2026-06-29

**Authors:** Rongying Xiao, Minghui Jin, Jixuan Heng, Feng Chang

**Affiliations:** 1 College of Agronomy, Xinyang Agriculture and Forestry University, Xinyang, China; 2 Chongqing Three Gorges Academy of Agricultural Sciences, Chongqing, China

**Keywords:** controlled-release urea, nitrogen management, nitrogen use efficiency, nutrient uptake, Oilseed rape (*Brassica napus* L.), soil inorganic nitrogen

## Abstract

Effective nitrogen management is essential for regulating nutrient uptake and improving productivity in oilseed rape (*Brassica napus* L.). However, mismatches between soil nitrogen availability and crop demand often limit nitrogen use efficiency (NUE). A two-year field experiment was conducted to compare nitrogen application strategies, including controlled-release urea (CRU), single-application urea (OU), split-application urea (TU), and treatments with a 25% reduction in nitrogen. The study evaluated their effects on soil inorganic nitrogen dynamics, biomass accumulation, nutrient uptake, yield components, and nitrogen use efficiency. The results showed that nitrogen release pattern strongly influenced soil nitrogen availability and crop performance. Both controlled-release urea and split-application enhanced biomass accumulation, nutrient uptake, and yield components compared with single-application urea. At an application rate of 240 kg N ha^-1^, overwintering dry matter increased by 19.9-28.2% in 2023 and 14.6-17.0% in 2024. Nitrogen accumulation increased by 17.6-32.0% in 2023 and 31.6-33.9% in 2024 under CRU240 and TU240 compared with OU240. Grain nitrogen accumulation showed even greater increases, reaching 115.7-149.3% in 2023 and 52.1-52.7% in 2024. Pod number also increased (18.7-19.4% in 2023 and 24.8-27.7% in 2024), along with nitrogen partial factor productivity (by 27.1-60.8%) and nitrogen recovery efficiency (by 16.0-25.7%) relative to OU. Additionally, CRU maintained higher levels of soil NH_4_^+^-N and NO_3_^--^N during the vegetative growth stage. A 25% reduction in nitrogen input decreased biomass and nutrient accumulation by 12-50%, although CRU180 and TU180 partially mitigated these reductions. Optimizing the timing of nitrogen release improves the synchronization between soil nitrogen supply and crop demand. Controlled-release urea performs comparably to split application while requiring fewer fertilizer applications. It also enhances the coordination of nutrient uptake, biomass accumulation, and nitrogen use efficiency, even under reduced nitrogen input. In conclusion, the reduced one-time application of controlled-release urea improved nutrient uptake coordination, nitrogen use efficiency, and crop productivity, indicating its potential as an effective nitrogen management strategy for winter oilseed rape production in the study region.

## 1 Introduction

Nitrogen fertilization is a key factor controlling crop yield and nutrient cycling in agricultural systems around the world. Oilseed rape (*Brassica napus* L.) is an important oilseed crop characterized by a relatively high nitrogen requirement per unit of seed yield rather than necessarily per unit land area. Quantitative nutrient requirement analysis has shown that approximately 46 kg N is needed to produce 1 t of seed, while agronomic recommendations often use about 50–60 kg N per additional ton of seed yield as a practical reference value (Ren et al., 2016). To sustain its rapid growth and efficient nutrient uptake, it requires a steady and well-synchronized supply of nitrogen from the soil throughout its life cycle (Łukowiak et al., 2025; Zhang Y.Y. et al., 2020). However, temporal mismatches between soil nitrogen availability and crop nitrogen demand remain a major constraint to nitrogen use efficiency and can negatively affect environmental sustainability in oilseed rape production (Villar et al., 2019). Urea is the most commonly used nitrogen fertilizer globally because it contains a high proportion of nitrogen and is relatively inexpensive. However, it undergoes rapid hydrolysis and subsequent nitrogen release, frequently resulting in significant nitrogen losses through processes such as volatilization, leaching, and denitrification, thereby reducing crop nitrogen recovery (Swify et al., 2023; Jia et al., 2021). To improve nitrogen synchronization, enhanced-efficiency fertilizers, such as controlled-release urea, along with optimized management practices like split application and reduced nitrogen inputs, have been increasingly adopted in oilseed rape production (Rahimitanha et al., 2022). Optimized nitrogen management, particularly appropriate adjustment of nitrogen rate and timing and the use of controlled-release nitrogen fertilizers, has been reported to improve nitrogen use efficiency in oilseed rape and, under specific conditions, maintain seed yield with reduced nitrogen inputs (Zhao et al., 2023; Hu et al., 2023).

Previous studies in oilseed rape (*Brassica napus* L.) have shown that controlled-release fertilizers can better synchronize nitrogen release with crop nitrogen demand by maintaining soil mineral nitrogen availability during the growth period. For example, a biochar-based controlled-release nitrogen fertilizer released nitrogen more slowly than conventional urea and increased soil NO**_3_^--^**N concentration, plant nitrogen uptake, nitrogen use efficiency, and crop growth in oilseed rape (Liao et al., 2020). Field studies on early-ripening rapeseed further demonstrated that controlled-release fertilizer improved plant growth, nutrient uptake, fertilizer use efficiency, and seed yield (Tian et al., 2016). More recently, controlled-release nitrogen blended with conventional nitrogen fertilizer was reported to maintain rapeseed yield under reduced nitrogen input by improving nitrogen uptake and utilization efficiency (Hu et al., 2023). In oilseed rape, moderate nitrogen reduction should be considered together with fertilizer type and cropping-system conditions. A two-year field study on direct-seeded rapeseed showed that a 25% reduction in nitrogen input, from 180 to 135 kg N ha**^-1^**, combined with 30%–50% controlled-release nitrogen blended with conventional nitrogen, maintained seed yield and improved nitrogen fertilizer utilization efficiency (Hu et al., 2023). Moreover, studies in oilseed rape and rape-rice rotation systems suggest that controlled-release nitrogen fertilizers may reduce reactive nitrogen losses, such as N**_2_**O emissions and nitrogen leaching, although these effects are site- and system-dependent (Liao et al., 2020; Ding et al., 2022). Despite these advances, most studies to date have primarily focused on either crop yield or nitrogen use efficiency, often treating them in isolation. As a result, the relationship between soil nitrogen supply dynamics and crop nutrient uptake processes remains insufficiently understood (Sentek et al., 2023). In particular, recent studies have shown that different urea-based fertilizers and application regimes can alter nitrogen release patterns, soil mineral nitrogen availability, crop nitrogen uptake, and dry matter accumulation in oilseed rape. For example, novel coated urea showed a prolonged nitrogen release period and better synchronization with oilseed rape nitrogen demand, resulting in higher aboveground dry matter, nitrogen accumulation, seed yield, and nitrogen use efficiency compared with conventional urea (Li et al., 2024). Furthermore, the absence of integrated experimental frameworks that directly compare controlled-release urea, single basal application, split application, and reduced nitrogen input scenarios has limited mechanistic understanding of how nitrogen supply and crop uptake are coordinated in oilseed rape systems.

The objective of this study was to determine how different urea types and application strategies affect soil nitrogen supply dynamics, nutrient uptake, dry matter accumulation, and nitrogen use efficiency in oilseed rape. The controlled-release urea tested in this study was an oilseed rape-specific CRU, developed according to the nutrient demand pattern of oilseed rape, and was selected because its nitrogen release profile is better synchronized with the temporal nitrogen requirement of oilseed rape than general slow- or controlled-release urea formulations. We hypothesized that this oilseed rape-specific CRU and split application of conventional urea would provide a more sustained nitrogen supply than a single basal application of conventional urea, thereby enhancing the coordination between soil nitrogen availability and crop nutrient demand, promoting dry matter accumulation and N, P, and K uptake, and helping maintain nitrogen use efficiency under a 25% reduction in nitrogen input. To test this hypothesis, a field experiment was conducted with a no-nitrogen control, single basal application of oilseed rape-specific CRU, single basal application of conventional urea, three-split application of conventional urea, and corresponding reduced-nitrogen treatments.

## 2 Materials and methods

### 2.1 Site information, experimental design, and management

**Figure 1:**
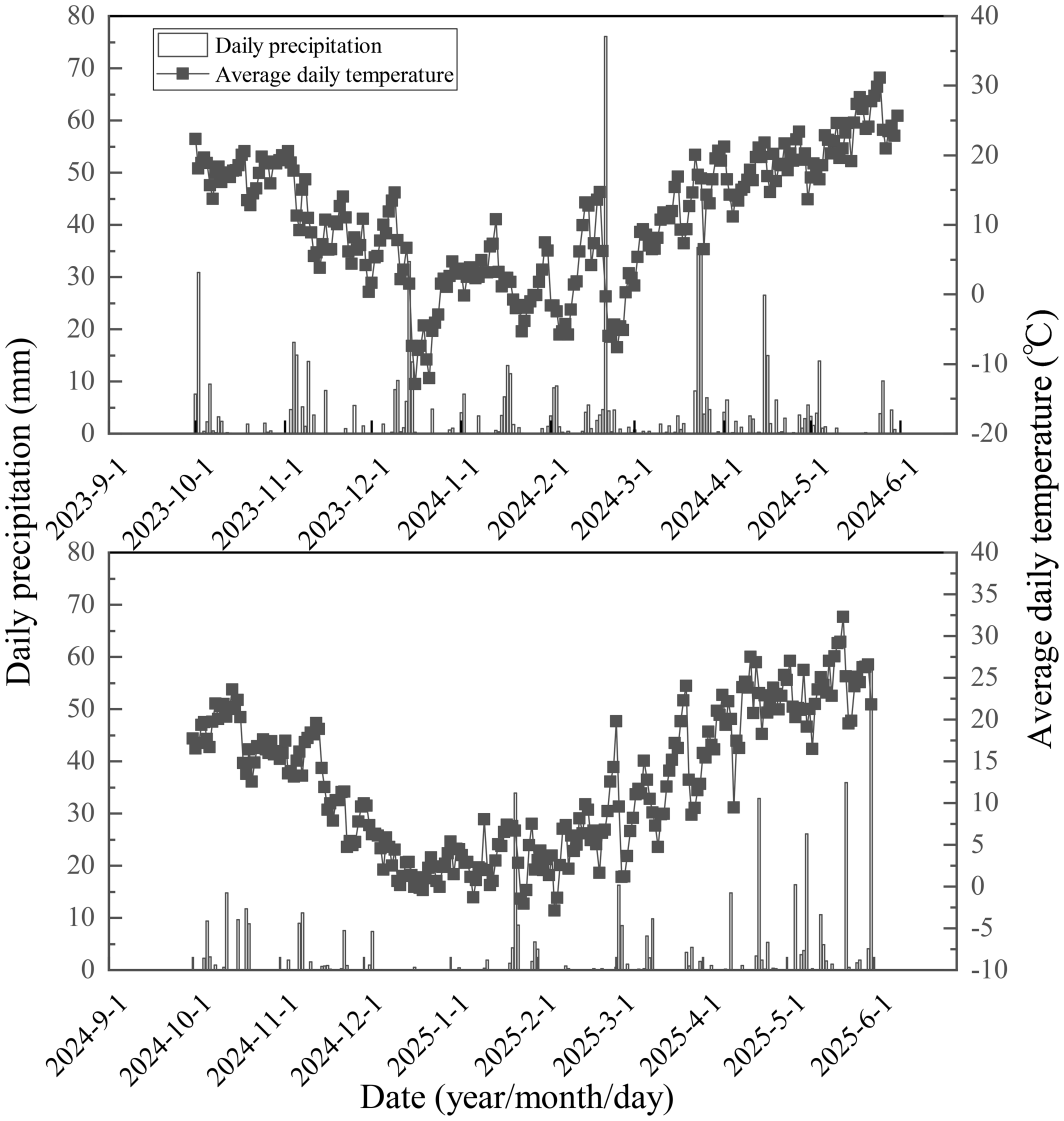
Daily precipitation and mean air temperature during the growth period of winter oilseed rape in 2023/2024 and 2024/2025.

The field experiment was conducted during 2023/2024 and 2024/2025 at Wudawan Village, Beixiangdian Township, Guangshan County, Xinyang City, Henan Province, China (N32°02′24′′, E114°54′36′′). For data on average daily temperatures and precipitation during the winter rapeseed growing seasons of 2023/2024 and 2024/2025, please refer to **Figure 1**. The meteorological data is sourced from NASA Power (Mehmet et al., 2023). The field soil (Anthrosols, IUSS Working Group WRB) was sandy loam with the following characteristics (0-20 cm layer): pH 5.24 (soil:water = 1:2.5), organic carbon 20.0 g·kg^-1^, total nitrogen (TN) 1.36 g·kg^-1^, available phosphorus 15.12 mg·kg^-1^, and available potassium 111.45 mg·kg^-1^. Based on local fertilization recommendations for winter oilseed rape and previous regional field studies, 240 kg N ha^-1^ was selected as the conventional nitrogen application rate, while 180 kg N ha^-1^ (25% reduction) was included to evaluate whether optimized nitrogen management strategies could maintain crop productivity and nitrogen use efficiency under reduced nitrogen input. Seven treatments were applied in the experiment:

1. N0: no nitrogen application.
2. CRU240: 240 kg N ha^-1^ as controlled-release urea, applied once as a basal fertilizer.
3. OU240: 240 kg N ha^-1^ as conventional urea, applied once as a basal fertilizer.
4. TU240: 240 kg N ha^-1^ as conventional urea, split into three applications (basal, seedling, and bolting stages).
5. CRU180: 180 kg N ha^-1^ (25% reduction) as controlled-release urea, applied once as a basal fertilizer.
6. OU180: 180 kg N ha^-1^ (25% reduction) as conventional urea, applied once as a basal fertilizer.
7. TU180: 180 kg N ha^-1^ (25% reduction) as conventional urea, split into three applications (basal, seedling, and bolting stages).

The experiment employed a randomized block design with three replicate plots per treatment, each measuring 20 m^2^ (4 m × 5 m). Nitrogen was supplied by controlled-release urea (CRU, 44% N) and conventional urea (46% N). The CRU used in this study was an oilseed rape-specific controlled-release urea containing 44% N. This product was developed according to the nitrogen demand pattern of oilseed rape, and its nitrogen release profile was designed to better match the temporal nitrogen requirement of oilseed rape than general slow- or controlled-release urea formulations. All treatments received identical fertilizer regimes except for nitrogen type. The P_2_O_5_ application rate was 90 kg·ha^-1^, the K_2_O application rate was 120 kg·ha^-1^, and the borax application rate was 15 kg·ha^-1^. The P_2_O_5_ fertilizer consisted of calcium superphosphate (12% P_2_O_5_), the K_2_O fertilizer consisted of potassium chloride (60% K_2_O), and the boron fertilizer consisted of borax (approximately 11% B). All phosphorus, potassium, and boron fertilizers were applied as a single basal dressing. Trenches between plots were 0.3 m wide, and trenches between replicate blocks were 0.4 m wide. A buffer zone was established around the trial perimeter, within which direct-seeded rapeseed was cultivated with half the fertilizer rate used in the main trial plots. The rapeseed variety used in this experiment was “Da Di 199” (*Brassica napus* L.). The preceding crop was rice. After the rice harvest, the rice straw was removed from the field, and the field was prepared prior to sowing. Rapeseed was sown on October 24, 2023, and October 27, 2024, respectively. The seeding rate was 5.25 kg·ha^-1^ (calculated based on an average thousand-seed weight of 3.55 g, equivalent to approximately 148,000 seeds·ha^-1^). The crops were grown under rain-fed conditions, and all treatment groups were managed uniformly using standard local weed, disease, and pest control measures.

### 2.2 Sampling and measurement

According to the BBCH staging system, plant and soil samples were collected at five key growth stages of rapeseed: the seedling stage (BBCH 12–19); the wintering stage (BBCH 19–21); the bolting stage (BBCH 30–39); the flowering stage (BBCH 60–65); and the maturity stage (BBCH 89). At the seedling, wintering, bolting, flowering, and maturity stages, rapeseed plants were sampled from each plot using a 0.36 m^2^ quadrat. At maturity, plants were separated into seeds, stems, and pods, and total biomass was calculated as the sum of their dry weights. For yield determination, a 10 m^2^ area per plot was manually harvested. Yield components, including siliques per plant, seeds per silique, and thousand-seed weight, were assessed from the 0.36 m^2^ subsamples. The number of viable pods on the main inflorescence and lateral branches of individual rapeseed plants was counted; pods containing more than five seeds were counted as viable pods, and their average was taken as the number of viable pods per plant. Thirty pods were randomly selected from each rapeseed plant, and the number of seeds inside each was counted; the average was used as the number of seeds per pod. Thousand-seed weight was determined by randomly weighing 1,000 air-dried rapeseed seeds using a thousand-seed scale.

All plant samples were oven-dried at 60 °C to constant weight. Nitrogen and phosphorus concentrations were determined following H_2_SO_4_-H_2_O_2_ digestion using a continuous flow analyzer (AA3, SEAL, Germany). Plant samples were digested using an H_2_SO_4_–H_2_O_2_ digestion procedure. dried and ground plant material was digested with concentrated H_2_SO_4_, followed by repeated addition of H_2_O_2_ until the digest became clear. The digestion was conducted on an electric digestion block at approximately 380 °C until complete digestion. Potassium concentration was measured after the same digestion procedure using a flame photometer (FP6410, Jingqi Instrument Ltd., Shanghai, China).

Soil samples were collected at the same growth stages using an S-shaped, five-point sampling method within each plot. Topsoil (0–20 cm) was composited, sieved through a 2 mm mesh, and stored at −20 °C for no longer than one month prior to analysis to minimize biological activity and changes in inorganic nitrogen concentrations. Soil inorganic nitrogen (NH_4_^+^-N and NO_3_^--^N) was extracted with 1 mol L^-1^ KCl and quantified using a continuous flow analyzer (AA3, SEAL, Germany). Soil inorganic nitrogen was extracted with 1 mol L^-1^ KCl at a soil ratio of 1:5, w/v. The suspension was shaken for 1 h, filtered, and the NH_4_^+^–N and NO_3_^–^N concentrations in the extract were determined.

### 2.3 Calculation of indicators

The following equations were used to calculate the relevant parameters (Bu et al., 2019):

Nitrogen partial efficiency (kg·kg^-1^) = N application area yield/Amount of N applied (1);
Nitrogen recovery efficiency (%) = (Total aboveground N uptake in the N application area - Total aboveground N uptake in N0 plot)/Amount of N applied × 100 (2);
Nitrogen agronomic efficiency (kg·kg^-1^) = (Yield in N applied area - Yield in N0 plot)/Amount of N applied (3);
Nitrogen internal efficiency (kg·kg^-1^) = Yield in N applied area/Total aboveground N uptake in the N applied area at maturity (4);
Nitrogen harvest index (%) = Grain N accumulation in the N applied area/Total aboveground N uptake in the N applied area at maturity × 100 (5).

### 2.4 Statistical analysis

Statistical analysis was performed using SPSS 26.0 (Chicago, IL, USA). One-way ANOVA followed by Duncan’s multiple range test was used to detect significant differences among treatments. Significant differences (*P < 0.05*) are indicated by different letters. Graphs were prepared using Origin 2021 (OriginLab, Northampton, USA).

## 3 Results

### 3.1 Effects of urea types on dry matter and nutrient accumulation in rapeseed

**Figure 2:**
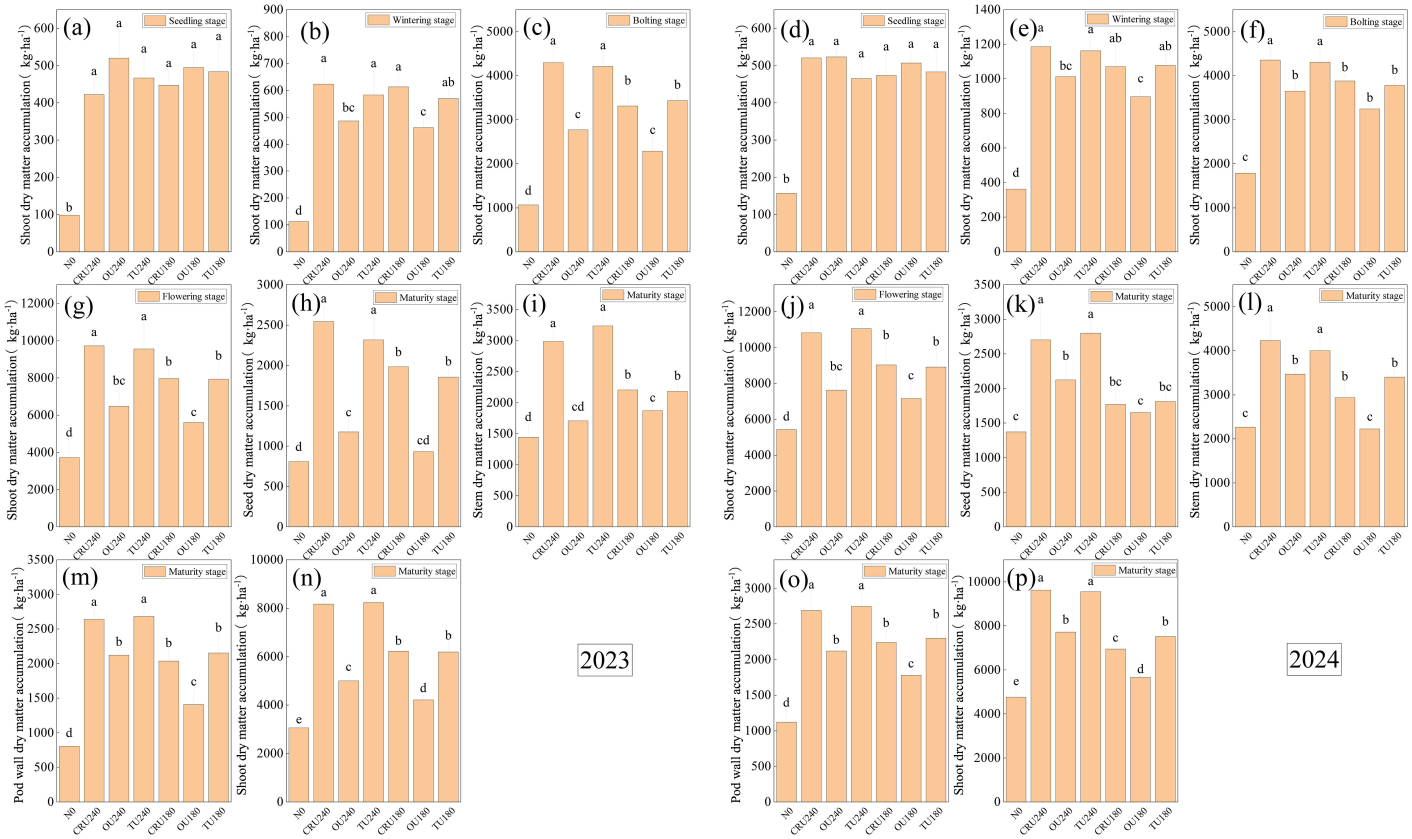
Effects of different urea types on dry matter accumulation during key growth stages of rapeseed. Treatments included: (1) N0, no nitrogen; (2) CRU240 and CRU180, controlled-release urea applied once basally; (3) OU240 and OU180, conventional urea applied once basally; (4) TU240 and TU180, conventional urea split into three applications (basal, seedling, and bolting). The numbers 240 and 180 indicate nitrogen application rates (kg N ha-1), The same as follows. In 2023, **(a–c, g, n)**, and **(n)** denote shoot dry matter accumulation at the seedling, wintering, bolting, flowering, and maturity stages, respectively; panels **(h)**, **(i)**, and **(m)** represent seed, stem, and pod wall dry matter accumulation at maturity. In 2024, **(d–f, j, p)** correspond to shoot dry matter accumulation at the seedling, wintering, bolting, flowering, and maturity stages, respectively; **(k, l, o)** represent seed, stem, and pod wall dry matter accumulation at maturity. Different lowercase letters within the same column indicate significant differences among nitrogen fertilizer treatments within the same year(P < 0.05), as determined by Duncan(n = 3), The same as follows.

Across the 2023 and 2024 growing seasons, aboveground dry matter accumulation (DMA) followed a typical sigmoidal growth pattern (**Figure 2**), with slow increases during the seedling and overwintering stages, rapid accumulation during bolting, and stabilization toward maturity. Nitrogen application significantly enhanced DMA at the seedling stage compared with the no-nitrogen control, whereas differences among urea types were not significant at this early stage. From overwintering onward, treatment effects became more pronounced. Controlled-release urea (CRU) and split application (TU) consistently resulted in greater biomass accumulation than conventional single basal urea (OU). For example, at 240 kg·ha^-1^, DMA at overwintering increased by 28.2% and 19.9% under CRU240 and TU240 in 2023, and by 17.0% and 14.6% in 2024, compared with OU240. Similar trends were observed under the reduced nitrogen rate (180 kg·ha^-1^).

A 25% reduction in nitrogen supply significantly limited biomass formation at later stages. At bolting, DMA under OU180, CRU180, and TU180 decreased by 29.6%, 21.2%, and 22.6% in 2023, and by 12.2%, 12.4%, and 14.1% in 2024, respectively, compared with their corresponding 240 kg·ha^-1^ treatment. At maturity, biomass partitioning differed among fertilizer regimes. Grain, stem, and pod dry matter were consistently higher under CRU and TU than under OU. For instance, compared with OU240, grain DMA increased by 116.3% and 97.1% under CRU240 and TU240 in 2023, and by 27.2% and 31.7% in 2024. However, nitrogen reduction markedly decreased dry matter allocation to reproductive organs, with pod biomass declining by 29.7%, 50.5%, and 24.7% in 2023, and approximately 19-20% in 2024, under OU180, CRU180, and TU180, respectively.

#### 3.1.1 Effects of different urea types on nitrogen accumulation during key growth stages of oilseed rape

**Figure 3:**
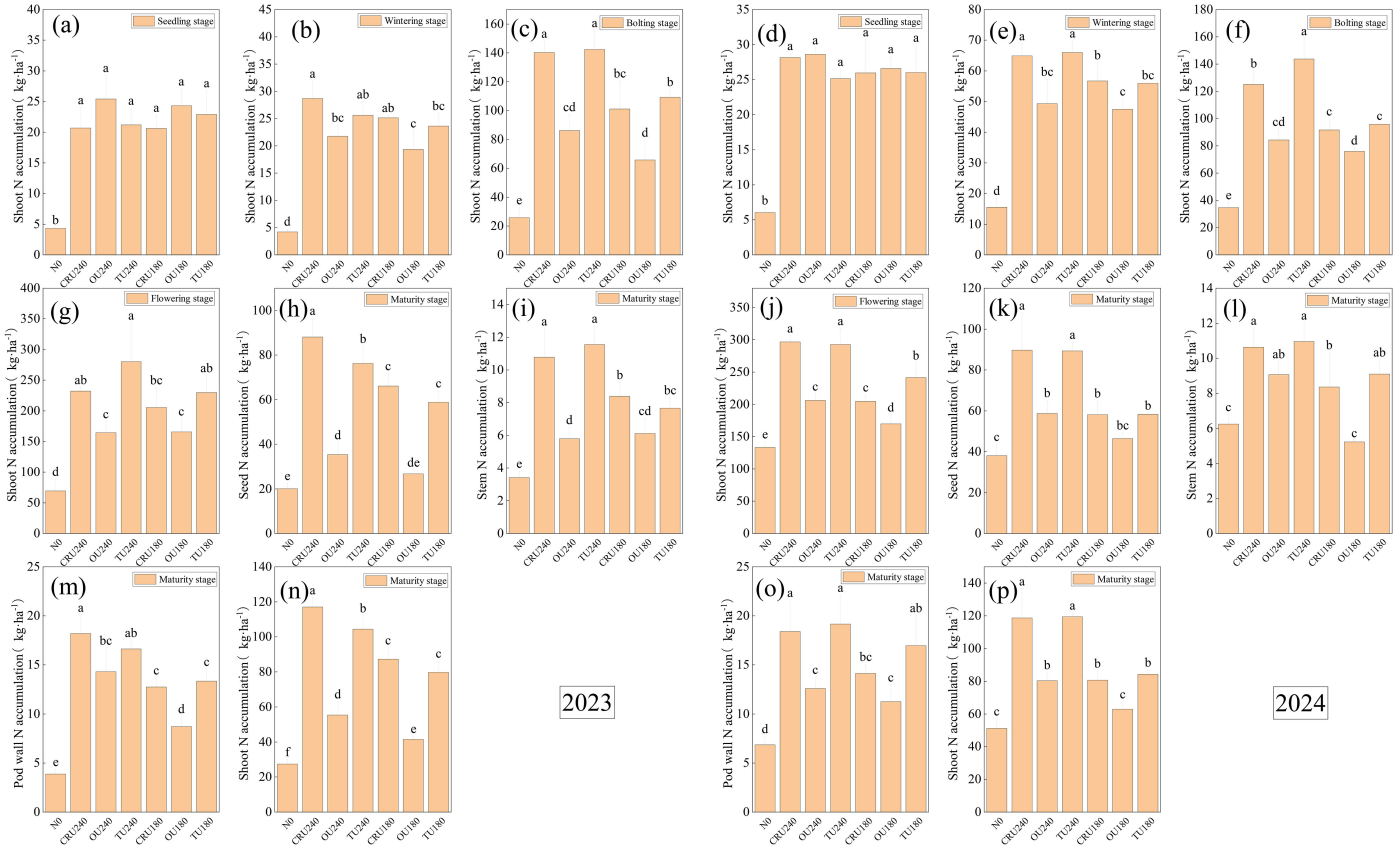
Effects of different urea types on nitrogen accumulation during key growth stages of rapeseed. The data are presented as the means/standard deviation (SD) of three replicates. In 2023, **(a–c, g, n)** denote shoot N accumulation at the seedling, wintering, bolting, flowering, and maturity stages, respectively; **(h, i, m)** represent seed, stem, and pod wall N accumulation at maturity. In 2024, panels **(d–f, j, p)** correspond to shoot N accumulation at the seedling, wintering, bolting, flowering, and maturity stages, respectively; **(k, l, o)** represent seed, stem, and pod wall N accumulation at maturity.

Aboveground nitrogen accumulation (NA) exhibited a similar sigmoidal pattern in both growing seasons (**Figure 3**). During early growth, accumulation was limited, then increased sharply at bolting, and gradually stabilized as plants reached maturity. At the seedling stage, all nitrogen treatments significantly increased NA compared with the N0, with no significant differences among urea types. From the overwintering stage onward, CRU and TU led to greater NA than OU. At a nitrogen rate of 240 kg·ha^-1^, overwintering NA under CRU240 and TU240 increased by 32.0% and 17.6% in 2023, and by 31.6% and 33.9% in 2024, relative to OU240. Similar trends were observed at 180 kg·ha^-1^.

Reductions in nitrogen supply significantly restricted NA during periods of rapid vegetative growth. At bolting, NA under OU180, CRU180, and TU180 decreased by 38.7%, 31.1%, and 30.3% in 2023, and by 36.6%, 10.9%, and 50.1% in 2024, respectively, compared with the corresponding 240 kg·ha^-1^ treatment. At maturity, nitrogen allocation to reproductive organs was strongly influenced by fertilizer type. NA in grains, stems, and pods was consistently higher under CRU and TU than under OU. Compared with OU240, grain NA increased by 149.3% and 115.7% in 2023, and by 52.7% and 52.1% in 2024 under CRU240 and TU240, respectively. In contrast, nitrogen reduction decreased NA in pods and other organs by 24-64% in 2023 and 12-30% in 2024.

#### 3.1.2 Effects of different urea types on phosphorus accumulation during key growth stages of oilseed rape

**Figure 4:**
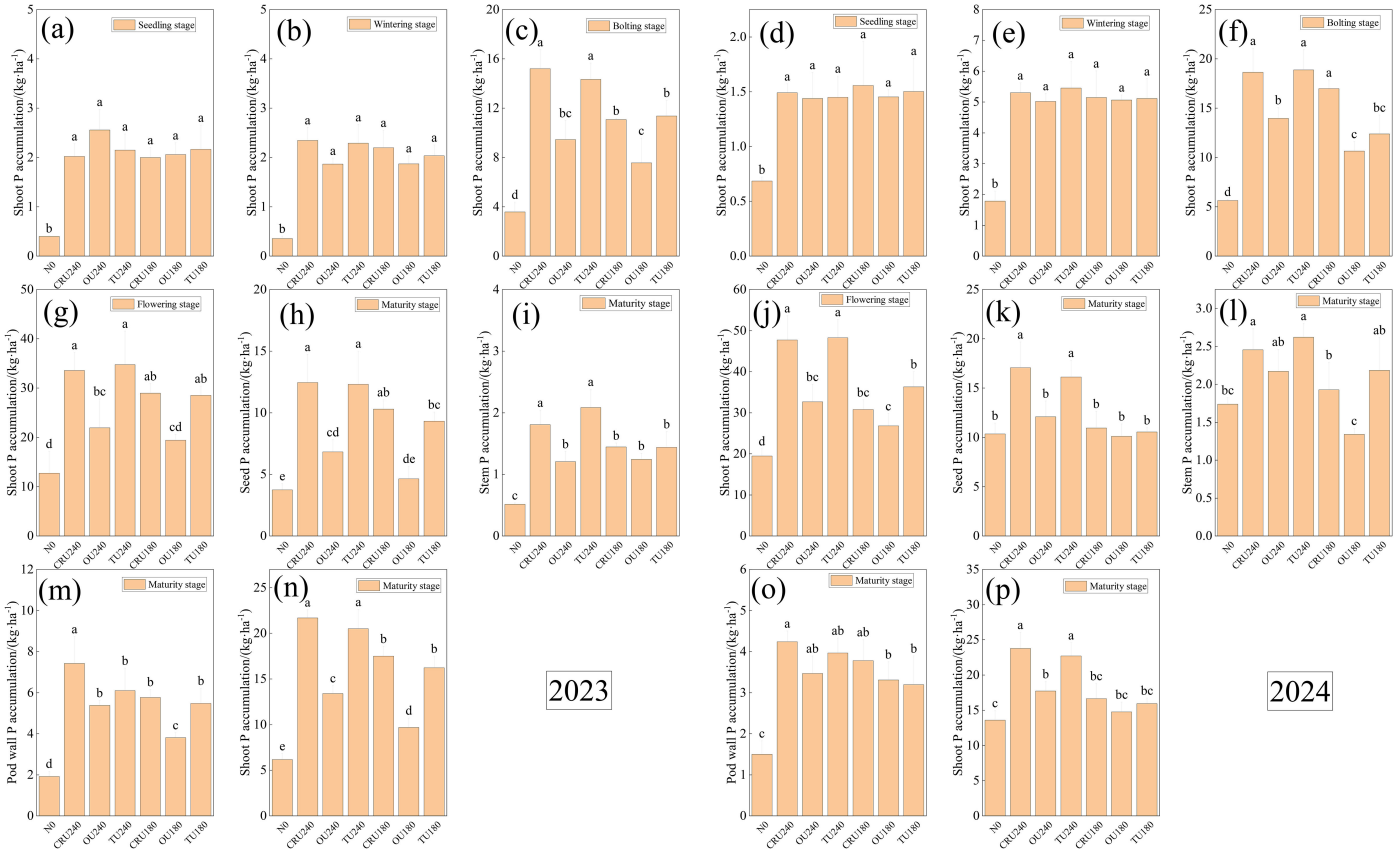
Effects of different urea types on phosphorus accumulation during key growth stages of rapeseed. In 2023, **(a–c, g, n)** denote shoot P accumulation at the seedling, wintering, bolting, flowering, and maturity stages, respectively; panels **(h)**, **(i)**, and **(m)** represent seed, stem, and pod wall P accumulation at maturity. In 2024, **(d–f, j, p)** correspond to shoot P accumulation at the seedling, wintering, bolting, flowering, and maturity stages, respectively; **(k, l, o)** represent seed, stem, and pod wall P accumulation at maturity.

Aboveground phosphorus accumulation (PA) also exhibited a sigmoidal pattern (**Figure 4**), with limited increases during early growth, rapid accumulation at bolting, and stabilization toward maturity. During the early stages, nitrogen application significantly enhanced PA compared with the N0 control, while differences among urea types were not significant. From bolting onward, CRU and TU consistently promoted higher PA than OU. At a nitrogen rate of 240 kg·ha^-1^, PA at bolting increased by 60.9% and 51.8% in 2023, and by 33.5% and 35.3% in 2024 under CRU240 and TU240, respectively, compared with OU240.

Nitrogen reduction significantly suppressed PA during flowering and reproductive development. At flowering, PA under reduced nitrogen decreased by 12.9-21.8% in 2023 and 21.9-54.9% in 2024 relative to the corresponding 240 kg·ha^-1^ treatments. At maturity, CRU and TU maintained higher phosphorus accumulation in grain, stems, and pods compared with OU. Grain PA increased by 82.6% and 80.4% in 2023, and by 33.5% and 35.3% in 2024 under CRU240 and TU240, respectively, relative to OU240. Nitrogen reduction significantly decreased pod PA by 11.5-41.5% in 2023 and 4.8-24.2% in 2024.

#### 3.1.3 Effects of different urea types on potassium accumulation during key growth stages of oilseed rape

**Figure 5:**
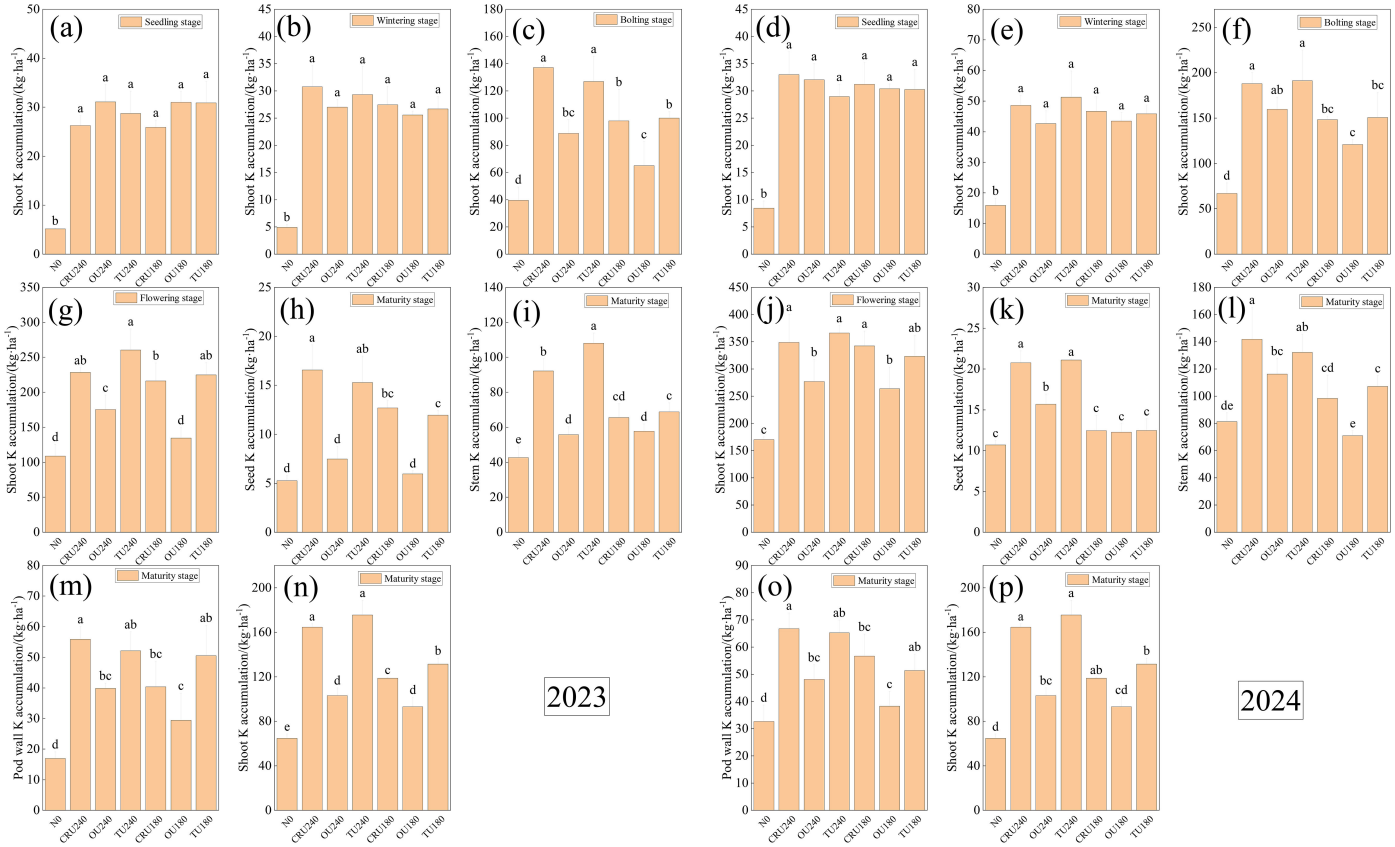
Effects of different urea types on potassium accumulation during key growth stages of rapeseed. In 2023, **(a-c,g, n)** denote shootK accumulation at the seeding, wintering bolting flowering, and maturity stages, respectively; panels **(h)**, **(i)**, **(m)** represent seed, stem and pod wall K accumulation at maturity. In 2024, **(d-f, j, p)** correspond to shoot K accumulation at the seeding, wintering bolting flowering, and maturity stages, respectively; **(k, l, o)** represent seed, stem and pod wall K accumulation at maturity.

Potassium accumulation (KA) followed a similar developmental pattern (**Figure 5**), with limited accumulation at early stages, rapid increases during bolting, and stabilization at maturity. Nitrogen fertilization significantly enhanced KA relative to the N0 control during early stages, with no significant differences among urea types. From bolting onward, CRU and TU consistently produced higher KA than OU. At a nitrogen rate of 240 kg·ha^-1^, KA at bolting increased by 54.5% and 43.0% in 2023, and by 17.6% and 19.6% in 2024 under CRU240 and TU240, respectively, compared with OU240.

Nitrogen reduction significantly limited KA during reproductive stages. At flowering, KA decreased by 5.7-30.2% in 2023 and 1.9-13.2% in 2024 under reduced nitrogen treatments relative to the 240 kg·ha^-1^ treatments. At maturity, CRU and TU promoted greater potassium allocation to grains, stems, and pods than OU. Grain KA increased by 121.7% and 104.5% in 2023, and by 32.5% and 34.5% in 2024 under CRU240 and TU240, respectively. Conversely, pod KA decreased by 3.3-38.5% in 2023 and 17.8-27.1% in 2024 under nitrogen reduction.

### 3.2 Effects of different urea types on rapeseed yield, yield components, and nitrogen use efficiency

Nitrogen application significantly increased seed yield and yield components (**Table 1**). At the same nitrogen rate, CRU and TU consistently produced more pods per plant than OU, and tended to increase seeds per silique. At 240 kg·ha^-1^, pod number increased by 18.7% and 19.4% in 2023, and by 24.8% and 27.7% in 2024 under CRU240 and TU240, respectively, compared with OU240. Similar advantages were observed at 180 kg·ha^-1^. There was no significant difference in grain yield between CRU and TU across the two growing seasons. At an application rate of 240 kg·ha^-1^, the yields of CRU240 and TU240 were 2603.3 and 2532.0 kg·ha in 2023, respectively, and 3016.6 and 2981.3 kg·ha^-1^ in 2024, respectively. This indicates that, in terms of maintaining grain yield, a single application of CRU is as effective as split applications of urea.

**Table 1:** Effects of different urea types on rapeseed yield, yield components, and nitrogen use efficiency.

Year	Treatment	Pod number per plant	Seeds per pod	1000-Seeds weight (g)	Yield (Kg·ha^-1^)	Nitrogen partial efficiency (kg·kg^-1^)	Nitrogen agronomic efficiency (kg·kg^-1^)	Nitrogen recovery efficiency (%)	Nitrogen Internal Efficiency(kg·kg^-1^)	Nitrogen Harvest Index (%)
2023	N0	119.9 ± 3.8d	18.99 ± 0.33c	3.76 ± 0.19a	671.87 ± 37.93d	/	/	/	33.76 ± 4.01b	12.49 ± 1.52a
	CRU240	324.7 ± 9.5a	22.97 ± 1.15a	3.81 ± 0.23a	2603.33 ± 79.65a	10.85 ± 0.33a	8.05 ± 0.23a	37.36 ± 3.03a	29.70 ± 2.17bc	3.95 ± 0.32d
	OU240	273.4 ± 6.9b	20.13 ± 0.46c	3.89 ± 0.12a	1619.48 ± 135.04b	6.75 ± 0.56c	3.95 ± 0.40c	11.69 ± 2.16c	46.42 ± 6.28a	8.61 ± 1.22c
	TU240	326.6 ± 6.2a	22.52 ± 1.31a	4.04 ± 0.23a	2531.97 ± 246.87a	10.55 ± 1.03a	7.75 ± 0.90a	32.09 ± 2.98ab	33.25 ± 1.63bc	4.36 ± 0.51d
	CRU180	281.9 ± 8.3b	22.29 ± 1.88ab	3.92 ± 0.07a	1781.9 ± 40.18b	9.90 ± 0.22ab	6.17 ± 0.33b	33.28 ± 5.01ab	27.04 ± 1.71c	5.04 ± 0.22d
	OU180	221.9 ± 2.1c	20.22 ± 0.48bc	4.05 ± 0.32a	1313.7 ± 16.46c	7.30 ± 0.09c	3.57 ± 0.30c	7.87 ± 0.99c	49.32 ± 2.78a	10.74 ± 0.56b
	TU180	282.5 ± 11.0b	20.86 ± 1.43abc	4.10 ± 0.21a	1703.13 ± 38.06b	9.46 ± 0.21b	5.73 ± 0.4b	29.12 ± 4.41b	29.1 ± 2.14bc	5.40 ± 0.29d
2024	N0	141.0 ± 10.8e	16.7 ± 0.8c	3.51 ± 0.42b	807.97 ± 44.51d	/	/	/	21.54 ± 3.50d	7.32 ± 0.66a
	CRU240	379.6 ± 15.9a	21.1 ± 2.7ab	4.19 ± 0.19a	3016.60 ± 175.65a	12.57 ± 0.73a	9.20 ± 0.58a	28.14 ± 4.96a	34.40 ± 5.70bc	3.79 ± 0.75d
	OU240	304.2 ± 6.5c	19.6 ± 0.8b	3.90 ± 0.15ab	2344.90 ± 155.04b	9.77 ± 0.65b	6.40 ± 0.58b	12.16 ± 3.30cd	40.24 ± 5.25ab	4.74 ± 0.51c
	TU240	388.4 ± 4.9a	22.7 ± 1.6a	3.98 ± 0.15a	2981.33 ± 139.43a	12.42 ± 0.58a	9.06 ± 0.72a	28.43 ± 2.33a	33.38 ± 0.14c	3.58 ± 0.22d
	CRU180	322.0 ± 6.1b	19.6 ± 1.5b	4.22 ± 0.08a	2424.73 ± 179.11b	13.47 ± 1.00a	8.98 ± 1.10a	16.38 ± 1.59bc	41.86 ± 2.99a	5.71 ± 0.75bc
	OU180	281.9 ± 9.4d	18.9 ± 1.9bc	3.93 ± 0.08ab	1665.77 ± 135.39c	9.25 ± 0.75b	4.77 ± 0.52c	6.51 ± 1.77d	35.86 ± 0.70abc	6.06 ± 0.26b
	TU180	327.6 ± 9.9b	20.6 ± 0.4ab	4.08 ± 0.37a	2464.10 ± 82.88b	13.69 ± 0.46a	9.20 ± 0.42a	18.42 ± 3.91b	42.27 ± 1.13a	5.52 ± 0.32bc
ANOVA analysis (*F* value)										
Year (Y)		282.81**	8.84**	0.25ns	162.27**	173.07**	108.95**	39.43**	0.02ns	87.05**
Treatment (T)		474.94**	8.41**	2.81*	193.50**	329.75**	189.35**	99.20**	16.58**	64.24**
Y×T		4.70**	0.96ns	1.54ns	4.75**	8.87**	7.03**	6.89**	17.02**	19.66**

Data are presented as means ± standard deviation (SD) of three replicates. Different lowercase letters within the same column indicate significant differences among nitrogen fertilizer treatments within the same year(P < 0.05), as determined by Duncan(n = 3). In the ANOVA analysis, * and ** indicate significant effects at the 5% and 1% levels respectively, and ns indicate no significant effect.

Nitrogen reduction significantly decreased pod number per plant by 15.2-23.2% in 2023 and 7.9-18.6% in 2024 compared with the corresponding 240 kg·ha^-1^ treatment. Nitrogen management also had a strong effect on nitrogen use efficiency (NUE). At identical nitrogen rates, CRU and TU improved both partial factor productivity and apparent nitrogen recovery efficiency relative to OU. At 240 kg·ha^-1^, partial factor productivity increased by 56.3-60.8% in 2023 and 27.1-28.6% in 2024 under CRU240 and TU240, while apparent nitrogen recovery efficiency increased by 16.0-25.7% compared with OU240. Similar improvements were observed under reduced nitrogen supply.

At the same nitrogen application rate, the Nitrogen Internal Efficiency (NIE) of the OU treatment was higher than that of the CRU and TU treatments; at a nitrogen application rate of 240 kg·ha^-1^, compared with CRU240 and TU240, the NIE of the OU240 treatment increased by 39.6–56.3% in 2023 and by 16.9–20.6% in 2024. Compared with the corresponding 240 kg·ha^-1^ treatments, reduced-nitrogen treatments generally increased Nitrogen Harvest Index (NHI), with NHI in the CRU and TU treatments increasing by 17.5–24.1% in 2023 and by 50.7–54.2% in 2024, indicating that nitrogen allocation to seeds increased under reduced nitrogen supply.

The results of the analysis of variance (ANOVA) indicated that year, treatment, and their interaction had significant effects (*P* < 0.01) on yield and most nitrogen use efficiency parameters. With the exception of 1000-Seeds weight, treatment was the primary source of variation for all traits; meanwhile, the significant year × treatment interaction observed in yield and nitrogen use efficiency parameters suggests that the effects of nitrogen management practices varied across different years.

### 3.3 Effects of different urea types on soil inorganic nitrogen content during key growth stages of rapeseed

Nitrogen fertilization significantly increased soil inorganic nitrogen (NH_4_^+^-N and NO_3_^--^N), with clear stage-dependent dynamics (**Table 2**). At the seedling stage, NH_4_^+^-N increased markedly with nitrogen rate, with the 240 kg·ha^-1^ treatments consistently exceeding the 180 kg·ha^-1^ treatments and the N0 control. CRU240 maintained the highest NH_4_^+^-N levels across both seasons. As growth progressed, NH_4_^+^-N declined in all treatments, but the decrease was more gradual under CRU. By flowering and maturity, NH_4_^+^-N concentrations converged across treatments.

**Table 2:** Effects of different urea types on changes in soil inorganic nitrogen content during key growth stages of rapeseed (mg/kg).

Year	Treatment	Seedling stage		Wintering stage		Bolting stage		Flowering stage		Maturity stage	
		NH_4_^+^	NO_3_^-^	NH_4_^+^	NO_3_^-^	NH_4_^+^	NO_3_^-^	NH_4_^+^	NO_3_^-^	NH_4_^+^	NO_3_^-^
2023	N0	3.31 ± 0.14d	4.21 ± 0.56d	4.53 ± 0.07f	2.81 ± 0.06e	5.72 ± 0.48d	2.77 ± 0.33d	3.87 ± 0.46a	13.21 ± 2.45a	3.69 ± 0.63a	13.28 ± 1.12a
	CRU240	35.41 ± 2.23a	25.24 ± 0.24a	15.82 ± 0.99a	20.39 ± 1.04a	13.63 ± 1.42a	17.4 ± 1.04a	4.61 ± 0.97a	14.84 ± 1.07a	4.48 ± 0.76a	14.54 ± 2.06a
	OU240	27.30 ± 3.39b	25.17 ± 2.3a	6.00 ± 0.54e	10.45 ± 1.01d	8.58 ± 1.11c	11.34 ± 1.65c	4.59 ± 0.6a	14.84 ± 1.53a	4.58 ± 0.85a	12.28 ± 1.60a
	TU240	15.73 ± 0.33c	21.16 ± 3.6b	15.61 ± 0.33a	11.43 ± 0.59cd	10.81 ± 1.07b	12.06 ± 1.59c	3.7 ± 0.35a	16.3 ± 1.07a	4.31 ± 0.74a	14.01 ± 1.10a
	CRU180	35.23 ± 3.12a	25.51 ± 0.41a	10.68 ± 0.83c	18.05 ± 0.5b	7.08 ± 0.41cd	15.01 ± 0.26b	4.19 ± 0.58a	14.87 ± 1.02a	4.96 ± 0.62a	12.10 ± 1.62a
	OU180	26.71 ± 2.27b	25.14 ± 3.09a	7.46 ± 1.20d	11.07 ± 1.05cd	9.04 ± 1.53bc	11.28 ± 0.92c	3.79 ± 0.43a	15.21 ± 1.97a	4.75 ± 0.97a	11.48 ± 1.38a
	TU180	13.43 ± 1.33c	8.78 ± 0.51c	12.24 ± 0.45b	12.72 ± 1.91c	10.98 ± 1.66b	11.46 ± 1.59c	3.7 ± 0.24a	14.28 ± 2.02a	3.73 ± 0.65a	12.35 ± 0.59a
2024	N0	12.55 ± 2.10e	11.23 ± 1.79d	6.02 ± 1.03d	11.05 ± 0.68d	10.20 ± 1.51c	10.96 ± 0.63d	4.39 ± 0.13a	15.38 ± 2.13a	6.50 ± 0.90a	8.11 ± 1.57a
	CRU240	46.56 ± 3.48a	44.21 ± 4.50a	16.59 ± 1.19a	27.16 ± 0.51a	18.98 ± 0.56a	22.85 ± 0.57a	5.07 ± 1.01a	16.77 ± 1.68a	8.78 ± 1.22a	9.16 ± 0.83a
	OU240	36.13 ± 2.83b	37.49 ± 3.68b	8.87 ± 1.01c	17.25 ± 3.10c	10.24 ± 0.88c	13.67 ± 1.61cd	4.21 ± 0.69a	17.97 ± 3.36a	7.79 ± 1.17a	8.01 ± 0.54a
	TU240	35.58 ± 4.04b	36.03 ± 1.51b	12.60 ± 1.49b	15.93 ± 0.11c	11.90 ± 1.64c	13.08 ± 2.07cd	4.2 ± 0.22a	18.48 ± 2.93a	7.62 ± 1.08a	9.29 ± 1.39a
	CRU180	28.17 ± 3.31c	23.94 ± 3.43c	13.47 ± 1.31b	20.46 ± 1.28b	16.51 ± 1.12b	17.81 ± 1.81b	4.60 ± 0.87a	17.35 ± 1.97a	8.30 ± 1.47a	8.86 ± 1.12a
	OU180	23.09 ± 2.38cd	24.16 ± 3.09c	6.86 ± 0.95cd	16.48 ± 1.94c	11.59 ± 2.26c	12.02 ± 0.24cd	4.33 ± 0.08a	16.33 ± 2.37a	7.03 ± 1.71a	8.94 ± 1.61a
	TU180	19.96 ± 2.39d	25.32 ± 2.12c	12.48 ± 1.26b	16.01 ± 0.34c	11.65 ± 0.64c	13.98 ± 1.86c	4.50 ± 0.82a	17.62 ± 1.83a	7.50 ± 1.42a	9.49 ± 1.72a
ANOVA analysis (*F* value)											
Year (Y)		62.75**	144.55**	4.57ns	180.20**	84.12**	65.67**	4.72ns	13.42**	99.88**	90.41**
Treatment (T)		102.09**	75.38**	101.75**	99.13**	25.24**	58.70**	1.50ns	1.20ns	1.59ns	1.45ns
Y×T		18.11**	15.59**	6.51**	3.91**	8.87**	6.06**	0.55ns	0.20ns	0.55ns	1.04ns

Data are presented as means ± standard deviation (SD) of three replicates. Different lowercase letters within the same column indicate significant differences among nitrogen fertilizer treatments within the same year (*P* < 0.05), as determined by Duncan’s test (n = 3). In the ANOVA analysis, ** indicate significant effects at the 5% and 1% levels respectively, and ns indicate no significant effect.

NO_3_^--^N exhibited a similar temporal pattern. Nitrogen application significantly elevated NO_3_^--^N at the seedling stage. Although levels declined during overwintering, CRU sustained comparatively higher NO_3_^--^N through bolting. Differences among treatments diminished toward maturity. Overall, CRU moderated the temporal decline in soil inorganic nitrogen and maintained greater nitrogen availability during vegetative growth.

The results of the analysis of variance (ANOVA) indicated that year, treatment, and their interaction had significant effects (*P* < 0.01) on soil NH_4_^+^-N and NO_3_^--^N contents during the seedling, wintering, and bolting stages. Year was the primary source of variation for most inorganic nitrogen parameters, whereas treatment effects were mainly detected during the early and middle growth stages. The significant year × treatment interactions observed for soil inorganic nitrogen contents indicate that the effects of different urea types on soil nitrogen dynamics varied between years. However, treatment and year × treatment interaction effects were generally not significant during the flowering and maturity stages, suggesting a convergence of soil inorganic nitrogen levels among treatments in the later growth period.

### 3.4 Coupling effects of different urea types on soil nitrogen supply and rapeseed nutrient uptake

**Figure 6:**
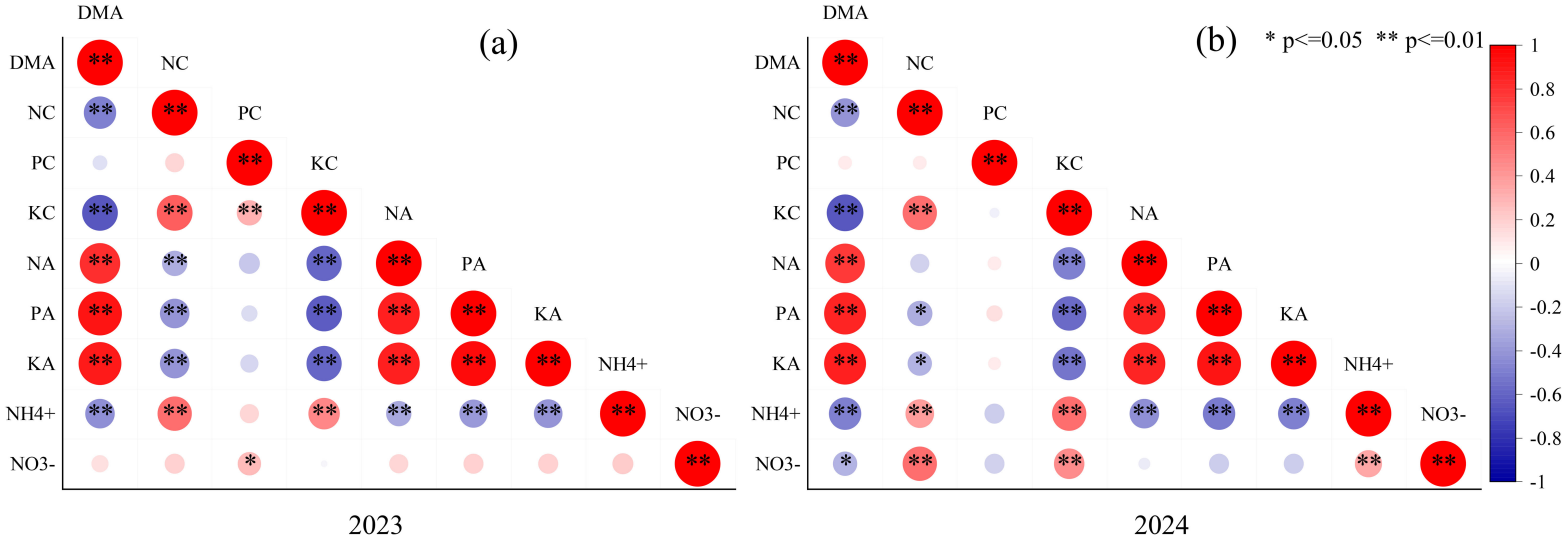
Correlation analysis between soil nitrogen supply and nutrient uptake in rapeseed in 2023 **(a)** and 2024 **(b)**. DMA, dry matter accumulation; NC, nitrogen content; PC, phosphorus content; KC, potassium content; NA, nitrogen accumulation; PA, phosphorus accumulation; KA, potassium accumulation; NH_4_^+^, ammonium nitrogen content; NO_3_^-^, Nitrate nitrogen content. * and ** indicate significant effects at the 5% and 1% levels respectively, as determined by Pearson correlation analysis (n = 3).

Correlation analysis revealed coordinated relationships between soil nitrogen supply and plant nutrient acquisition (**Figure 6**). Dry matter accumulation (DMA) was significantly and positively correlated with nitrogen, phosphorus, and potassium accumulation (NA, PA, and KA), indicating synchronized biomass production and nutrient uptake. Soil NH_4_^+^-N showed strong positive correlations with plant nitrogen content (NC) and NA, with higher coefficients than those for NO_3_^--^N, suggesting a closer association between ammonium availability and nitrogen assimilation. Although NO_3_^--^N was also positively correlated with NA and DMA, these relationships were comparatively weaker.

In some treatments, soil inorganic nitrogen was negatively correlated with plant potassium content (KC). Similar relationships have been reported under conditions of elevated ammonium availability, where NH_4_^+^ may compete with K^+^ uptake and influence plant ion balance (Coskun et al., 2017). However, because the present study was based on correlation analysis, the underlying mechanisms remain speculative and require further investigation. In some treatments, soil inorganic nitrogen was negatively correlated with plant potassium content (KC), suggesting potential antagonistic interactions between elevated mineral nitrogen levels and potassium uptake or allocation. Overall, these results indicate that optimizing the form and release pattern of nitrogen is critical for maintaining coordinated nutrient uptake and biomass accumulation in rapeseed.

## 4 Discussion

The synchronization between soil nitrogen release and crop demand fundamentally determines nitrogen uptake efficiency and subsequent biomass formation. Conventional urea releases most of its nitrogen shortly after application, often resulting in a mismatch between soil nitrogen supply and crop demand later in the season. This asynchronous release accelerates nitrogen losses through volatilization and leaching, thereby reducing crop nitrogen recovery, particularly in long-duration crops such as oilseed rape (Heuermann et al., 2021; Wang et al., 2021). In contrast, controlled-release urea (CRU) and optimized application strategies such as split applications extend the period of effective nitrogen availability, better aligning nitrogen supply with plant demand across growth stages (Zhu et al., 2020). Consistent with regional studies showing improved nitrogen synchronization and enhanced soil nitrogen pools under CRU compared with conventional urea (Yang et al., 2021), our results demonstrate that CRU maintained higher NH_4_^+^-N and NO_3_^--^N levels during overwintering and bolting, stages critical for nutrient uptake and biomass accumulation. Additionally, mixed CRU treatments have been shown elsewhere to sustain soil nitrogen and promote microbial activity, further supporting sustained nitrogen supply and crop uptake (Zhang L. et al., 2023).

Beyond release kinetics, controlled-release formulations may also regulate soil biochemical processes. Studies have reported that controlled-release nitrogen fertilizers not only extend soil nitrogen availability but also influence soil microbial dynamics and enzyme activities, further sustaining nitrogen supply and plant uptake (Zhang J.J. et al., 2023). For example, biochar-based controlled-release N fertilizers significantly improved soil microbial biomass nitrogen and enzyme activities, enhancing nitrogen availability during late growth stages and delaying vegetative senescence in oilseed rape. These effects supported greater nitrogen uptake and increased grain yield under equivalent nitrogen inputs (Liao et al., 2020; Khan et al., 2022). Furthermore, regional trials of CRU tailored for winter oilseed rape demonstrated consistent increases in both seed yield and nitrogen use efficiency compared with conventional urea across diverse climatic conditions, highlighting the broad agronomic potential of CRU strategies in long-duration crops (Zhang K.P. et al., 2020). Treatment responses varied across growing seasons. The differences in temperature and precipitation shown in **Figure 1** may have influenced soil nitrogen dynamics, crop nitrogen uptake, and the release characteristics of CRU, thereby leading to differences in grain nitrogen accumulation, yield components, and nitrogen use efficiency.

Efficient biomass formation in oilseed rape depends not only on nitrogen but also on the coordinated uptake of phosphorus and potassium. The strong positive coupling dry matter accumulation (DMA), nitrogen, phosphorus, and potassium accumulation (NA, PA, and KA) indicates that biomass formation is driven by multi-nutrient assimilation rather than nitrogen supply alone (Wu et al., 2019). Previous studies have highlighted that improving nitrogen availability through slow-release or blended fertilizers can enhance overall nutrient uptake and yield in rapeseed (Tian et al., 2016). Under CRU and split application regimes, sustained soil nitrogen likely maintained active root nutrient uptake during later growth stages, resulting in higher P and K accumulation compared with conventional urea (Ma et al., 2023). The relatively stronger association of soil NH_4_^+^-N with plant nitrogen accumulation, compared with NO_3_^--^N, suggests that a stable ammonium supply is particularly important for efficient assimilation. This supports previous findings that different nitrogen forms influence uptake kinetics and assimilation pathways (Zhang et al., 2019).

The benefits of controlled and slow nitrogen release are not limited to rapeseed. Studies in cereals have shown that slow- or controlled-release nitrogen fertilizers improve dry matter and nitrogen accumulation by better synchronizing soil nitrogen supply with crop demand, reducing excessive early nitrogen peaks, and minimizing growth-limiting late nitrogen deficits (Zhao et al., 2025). Collectively, these cross-species observations indicate that moderated nitrogen release represents a general regulatory mechanism that enhances nutrient assimilation efficiency during critical growth stages.

Optimized nitrogen management improved pod number, grain nitrogen accumulation, and nitrogen use efficiency. Similar observations have been reported previously; CRU applications increased the yield and nitrogen recovery efficiency of rapeseed relative to conventional urea treatments by enhancing soil inorganic nitrogen retention and nutrient uptake (Hu et al., 2023). Field trials have shown that CRU can increase rapeseed yield by 7-20% and improve nitrogen recovery efficiency by 12-27% compared with conventional urea, while achieving similar or better outcomes than split urea applications (Liu et al., 2019). The larger increase in grain N accumulation observed in 2023 than in 2024 may be associated with differences in precipitation and temperature patterns between growing seasons, which could influence soil nitrogen availability, crop nitrogen uptake, and the effectiveness of controlled-release nitrogen supply (Liu et al., 2019). Meta-analyses across staple crops have further confirmed that CRU improves grain yield, total nitrogen uptake, and various nitrogen use efficiency indices relative to conventional urea (Chen et al., 2020). These benefits are attributed to prolonged nitrogen availability, which supports critical physiological processes during reproductive growth.

However, a 25% reduction in total nitrogen input constrained nutrient accumulation and yield components, indicating that reduced input without adequate synchronization may limit crop performance (Kubar et al., 2022). This trade-off aligns with findings that optimized nitrogen reduction can maintain yield only when combined with improved fertilizer formulations or adjusted application timing (Quemada et al., 2013).

Convergent evidence from multi-environment trials further demonstrates that CRU improves yield and multiple nitrogen use efficiency indices across crops, with average increases in grain yield, nitrogen agronomic efficiency, recovery efficiency, and physiological nitrogen efficiency relative to conventional urea. These improvements are attributed to its slow-release characteristics, which better align with crop demand patterns (Zhang et al., 2022). Field experiments combining controlled-release nitrogen with conventional nitrogen have also shown that such hybrid fertilization strategies can maintain high yield levels and improve nitrogen use efficiency under reduced total nitrogen application, offering an effective balance between output and input reduction (Fan et al., 2021).

The integrative framework established here demonstrates that nitrogen form and release patterns act as key regulatory drivers that coordinate soil nutrient dynamics with plant physiological demand, supporting sustainable oilseed rape production. Controlled-release and split application strategies improved nutrient uptake coordination, biomass accumulation, and nitrogen use efficiency by maintaining soil nitrogen availability across different growth stages (Zhang K.P. et al., 2020). These results suggest that enhanced-efficiency fertilizers and optimized application strategies may improve the coordination between soil nitrogen availability and crop nutrient uptake, thereby contributing to reduced nitrogen losses and maintained yield. Such approaches align with broader efforts to improve fertilizer management in intensive cropping systems, reducing environmental risks and enhancing resource use efficiency without compromising productivity (Liu et al., 2025).

It should be noted that this study primarily assessed nutrient status based on nutrient accumulation and uptake characteristics. The study did not employ critical nutrient concentration models or nutrient dilution curves; therefore, the results cannot directly determine whether the nutrient status of crops at different growth stages was above, equal to, or below the critical sufficiency threshold. Furthermore, although the observed relationships between soil nitrogen availability, nutrient uptake, and crop performance suggest that nitrogen supply and plant demand are better synchronized under optimized urea management, the underlying physiological and biochemical mechanisms have not been directly investigated and thus require further validation.

Beyond yield and nutrient use, previous studies have reported that controlled-release nitrogen fertilizers may reduce nitrogen losses and environmental risks under certain production systems. For example, reduced rates of controlled-release nitrogen application have been shown to decrease nitrogen losses through runoff while maintaining or improving nitrogen use efficiency (He et al., 2025). Although nitrogen loss pathways were not directly measured in the present study, these findings from the literature suggest that optimized controlled-release nitrogen strategies may provide environmental benefits in addition to agronomic advantages.

## 5 Conclusions

Nitrogen type and release pattern regulate the temporal distribution of soil inorganic nitrogen and its synchronization with crop demand. Controlled-release urea (CRU) and split urea application (TU) maintained higher NH_4_^+^-N and NO_3_^--^N availability during the overwintering and bolting stages, strengthening the coordination between soil nitrogen supply and plant nutrient uptake. This improved synchronization promoted dry matter accumulation and enhanced the coupled uptake of nitrogen, phosphorus, and potassium compared with the single basal urea application.

At the same nitrogen rate, CRU and TU increased Pod number per plant by 18.8–27.7%, nitrogen partial factor productivity by 27.1–60.7%, and nitrogen recovery efficiency by 16.0–25.7 percentage points, compared with conventional urea. Although a 25% reduction in nitrogen input constrained biomass and nutrient accumulation, CRU180 and TU180 partly alleviated these limitations, maintaining relatively stable yield and nutrient uptake efficiency. Given its comparable agronomic performance and lower fertilization frequency, CRU represents a more efficient nitrogen management strategy. Overall, optimizing nitrogen release characteristics, particularly through controlled-release urea, provides a practical pathway to improve nitrogen use efficiency and sustain oilseed rape productivity under reduced nitrogen input. Given its comparable agronomic performance and lower fertilization frequency, CRU showed considerable potential as an efficient nitrogen management strategy under the conditions of the present study. Overall, optimizing nitrogen release characteristics, particularly through controlled-release urea, may provide a practical approach to improving nitrogen use efficiency and sustaining oilseed rape productivity under reduced nitrogen input. However, further studies across diverse soil types, climatic conditions, and production systems are needed to validate the broader applicability of these findings.

## Data Availability

The raw data supporting the conclusions of this article will be made available by the authors, without undue reservation.
